# Machine learning applied in maternal and fetal health: a narrative review focused on pregnancy diseases and complications

**DOI:** 10.3389/fendo.2023.1130139

**Published:** 2023-05-19

**Authors:** Daniela Mennickent, Andrés Rodríguez, Ma. Cecilia Opazo, Claudia A. Riedel, Erica Castro, Alma Eriz-Salinas, Javiera Appel-Rubio, Claudio Aguayo, Alicia E. Damiano, Enrique Guzmán-Gutiérrez, Juan Araya

**Affiliations:** ^1^ Departamento de Bioquímica Clínica e Inmunología, Facultad de Farmacia, Universidad de Concepción, Concepción, Chile; ^2^ Departamento de Análisis Instrumental, Facultad de Farmacia, Universidad de Concepción, Concepción, Chile; ^3^ Machine Learning Applied in Biomedicine (MLAB), Concepción, Chile; ^4^ Departamento de Ciencias Básicas, Facultad de Ciencias, Universidad del Bío-Bío, Chillán, Chile; ^5^ Instituto de Ciencias Naturales, Facultad de Medicina Veterinaria y Agronomía, Universidad de Las Américas, Santiago, Chile; ^6^ Millennium Institute on Immunology and Immunotherapy, Santiago, Chile; ^7^ Departamento de Ciencias Biológicas, Facultad de Ciencias de la Vida, Universidad Andrés Bello, Santiago, Chile; ^8^ Departamento de Obstetricia y Puericultura, Facultad de Ciencias de la Salud, Universidad de Atacama, Copiapó, Chile; ^9^ Departamento de Obstetricia y Puericultura, Facultad de Medicina, Universidad de Concepción, Concepción, Chile; ^10^ Cátedra de Biología Celular y Molecular, Departamento de Ciencias Biológicas, Facultad de Farmacia y Bioquímica, Universidad de Buenos Aires, Buenos Aires, Argentina; ^11^ Laboratorio de Biología de la Reproducción, Instituto de Fisiología y Biofísica Bernardo Houssay (IFIBIO-Houssay)- CONICET, Universidad de Buenos Aires, Buenos Aires, Argentina

**Keywords:** machine learning, artificial intelligence, pregnancy diseases, pregnancy complications, adverse perinatal outcomes

## Abstract

**Introduction:**

Machine learning (ML) corresponds to a wide variety of methods that use mathematics, statistics and computational science to learn from multiple variables simultaneously. By means of pattern recognition, ML methods are able to find hidden correlations and accomplish accurate predictions regarding different conditions. ML has been successfully used to solve varied problems in different areas of science, such as psychology, economics, biology and chemistry. Therefore, we wondered how far it has penetrated into the field of obstetrics and gynecology.

**Aim:**

To describe the state of art regarding the use of ML in the context of pregnancy diseases and complications.

**Methodology:**

Publications were searched in PubMed, Web of Science and Google Scholar. Seven subjects of interest were considered: gestational diabetes mellitus, preeclampsia, perinatal death, spontaneous abortion, preterm birth, cesarean section, and fetal malformations.

**Current state:**

ML has been widely applied in all the included subjects. Its uses are varied, the most common being the prediction of perinatal disorders. Other ML applications include (but are not restricted to) biomarker discovery, risk estimation, correlation assessment, pharmacological treatment prediction, drug screening, data acquisition and data extraction. Most of the reviewed articles were published in the last five years. The most employed ML methods in the field are non-linear. Except for logistic regression, linear methods are rarely used.

**Future challenges:**

To improve data recording, storage and update in medical and research settings from different realities. To develop more accurate and understandable ML models using data from cutting-edge instruments. To carry out validation and impact analysis studies of currently existing high-accuracy ML models.

**Conclusion:**

The use of ML in pregnancy diseases and complications is quite recent, and has increased over the last few years. The applications are varied and point not only to the diagnosis, but also to the management, treatment, and pathophysiological understanding of perinatal alterations. Facing the challenges that come with working with different types of data, the handling of increasingly large amounts of information, the development of emerging technologies, and the need of translational studies, it is expected that the use of ML continue growing in the field of obstetrics and gynecology.

## 1 Introduction

Pregnancy is a physiological process that provides all conditions for normal fetus growth and subsequent birth. Due to certain circumstances, a seemingly normal pregnant woman starts with physiological disorders that can trigger pregnancy diseases (e.g. gestational diabetes mellitus and preeclampsia) or other perinatal complications (e.g. stillbirth, cesarian section, macrosomia and respiratory distress). The search for new strategies for early diagnosis, screening and risk determination could reduce the severity of these alterations and also the negative impact in both mother’s and offspring’s health. Interestingly, in recent years, machine learning (ML) has been used to find solutions for these problems.

ML corresponds to a wide variety of methods that use mathematics, statistics and computational science to learn from multivariate data. By means of pattern recognition performed on various measured variables, different algorithms are able to find correlations, often hidden to the human eye, and perform accurate predictions about different conditions, such as the belonging of an individual to a certain group or class, or the concentration of a particular biomarker in a sample of interest.

Multivariate methods (i.e. those employed to analyze the behavior of multiple variables simultaneously) have been used for several decades to solve problems in different areas of knowledge, such as psychology, economics, biology, chemistry, etc. However, in the clinical field these tools have begun to penetrate only recently. Remarkably, the use of these tools has received different names throughout history depending on the area of application, i.e. psychometrics in psychology, biometrics in biology, chemometrics in chemistry, etc. In the last years it has become popular to address to these methods as artificial intelligence, ML, data mining, or in a more general sense, data science. The boundaries between the scopes of these different terms are still a subject of debate, and several different opinions and definitions can be found in specialized literature (1, 2). However, unconcerned of this debate, it seems that ML has been the preferred name used in healthcare-related studies, therefore that will be the term used in this manuscript.

One of the most common applications of ML in biomedicine is the detection or prediction of particular pathological conditions (3). It seems logical that in pregnancy the focus has also been in diagnostics (4, 5). However, as it has been evidenced in different disciplines, ML can also be used for other purposes, such as identification of important variables in a system or process, correlation analysis, data management and extraction, noise removal, dimensionality reduction, among others (6, 7). Given the success ML has had in other areas of science, we wondered how far it has penetrated into the field of obstetrics and gynecology. In this review we propose to describe the state of art regarding the use of ML in the context of pregnancy diseases and complications, including its capability for early diagnosis, screening and risk determination, and also other applications of this versatile tool.

## 2 Methodology

### 2.1 Type of study and search strategy

This is a narrative review. Publications regarding the use of ML in maternal and fetal health were searched in different databases, including PubMed, Web of Science and Google Scholar. Seven subjects of interest were considered as representative conditions of the vast domain of obstetrics and gynecology, due to their prevalence and clinical relevance (8): gestational diabetes mellitus, preeclampsia, perinatal death, spontaneous abortion, preterm birth, cesarean section, and fetal malformations.

### 2.2 Information synthesis

The papers main results were summarized in tables, comprising input, ML technique and output. Tables should be understood as follows: each table is associated with a specific pregnancy disease or complication, as stated on the table’s title. For every table, each row refers to a particular study. For each reference (first column), the input, the ML technique and the output (third, fourth and fifth columns, respectively) are directly linked to the ML application (second column) of that study. Most of the tabulated information is written and further extended in the text related to each table.

### 2.3 Manuscript organization

This manuscript is organized as follows: section 3 gives a general overview on ML-related definitions and concepts, section 4 describes different ML applications in the context of pregnancy diseases and complications, addressed from the highest to the lowest prevalence, section 5 discusses the current state and future challenges in the field, and section 6 rounds off with a brief conclusion.

## 3 ML: definitions and concepts

ML models can have varied purposes. The most typical one is early detection, but they can also be used for alternative screening, risk estimation, correlation assessment, biomarker discovery, among other possible applications.

In very simple terms, the development of a ML model requires three main parts: the input, the ML technique and the output.

The input is the data that is used to build the ML model. It consists of samples (usually in the biomedical field, the subjects) and variables, which can be very diverse. There is discrete data, e.g. the information retrieved from questionaries; the clinical and biochemical data found in physical and electronic health records (EHR); and the metabolites, peptides/proteins, transcripts or genes identified as relevant in omics studies. Likewise, there is continuous data, e.g. the traces obtained by Doppler ultrasonography, electrohysterography (EHG) or cardiotocography (CTG); and the images recorded by ultrasonography, computed tomography (CT) or echocardiography. The type of data determines what kind of pretreatment has to be performed prior to ML analysis, an aspect that is described in detail elsewhere (9, 10).

The selection of the ML technique depends on the purpose of the study. Non-supervised techniques are used to explore the data, i.e. to assess if there is any spontaneous clustering or correlation between samples and/or variables. Typical examples of non-supervised techniques are principal component analysis (PCA) and K-means. In contrast, supervised techniques are used to predict a property. In the ML field, the word “prediction” refers to the forecast of future behaviors or unobserved outcomes (11). In particular, classification ML techniques allow to predict a class or category, e.g. healthy or diseased; whereas regression ML techniques allow to predict a continuous quantity, e.g. the concentration of a specific biomarker. Moreover, supervised techniques can be linear or non-linear, depending on the nature of the mathematical function that underlies the classification or regression task. The most common linear classifiers are logistic regression (LR) and linear discriminant analysis (LDA), while some examples of linear regression techniques are linear regression and partial least squares (PLS). On the other hand, random forest (RF), support vector machines (SVM) and neural networks (NN) are classical examples of non-linear ML techniques that allow to perform both classification and regression analyses.

The output is the result of having applied the ML model. The most common outputs are those that account for the model predictive performance. In classification, the performance is typically expressed using parameters such as sensitivity, specificity, accuracy and area under the receiver operating characteristic curve (AUC). In regression, other parameters, such as mean absolute error and root mean squared error (RMSE), are used. These and other performance metrics are well described in literature (12, 13). It is important to mention that the aforementioned metrics can be calculated in different stages of the model’s development: training, internal validation and external validation. The ideal situation is that the model is tested in all the three stages, to ensure it will be accurate and useful in different populations. This idea has been discussed in greater depth by other authors (14, 15). Another very common output is variable importance. This information allows to identify the variables that contribute the most to predict the property under study, which is useful to identify new biomarkers for a certain condition. There are other possible outputs, depending on the ML application. They are addressed and discussed throughout section 4.

## 4 ML in pregnancy diseases and complications: applications

### 4.1 Pregnancy diseases

#### 4.1.1 Gestational diabetes mellitus

The American Diabetes Association defines gestational diabetes mellitus (GDM) as a “diabetes diagnosed in the second or third trimester of pregnancy that was not clearly overt diabetes prior to gestation” (16). This disease has been related to several negative outcomes on maternal and fetal health. In the short-term, it increases the risk of pre-eclampsia, preterm delivery, macrosomia and clinical neonatal hypoglycemia; and in the long-term, of maternal prediabetes, maternal diabetes, offspring obesity and offspring impaired fasting glucose (17).

ML has been applied in GDM research, for diverse purposes (**Table 1**).

**Table 1:** ML applications in GDM research.

Reference	ML application	Input	ML technique	Main output
(18)	Disease prediction	Biochemical markers	Light GB	AUC = 99.83%, Se = 92.5% and Sp = 99.2%
(19)	Disease prediction	Clinical and biochemical factors	Bayesian LR	AUC = 0.766, Ac = 0.64, Se = 0.66 and Sp = 0.75
(20)	Disease prediction	Electronic health records	DNN	AUC = 0.80, Se = 63%, Sp = 82% and YI = 0.45
(21)	Disease prediction	Electronic health records	GB	AUC = 0.850 and AUPR = 0.324
(22)	Disease prediction	Clinical parameters	RF	Ac = 77.53%
(23)	Disease prediction, independent of diagnostic criteria	Clinical and biochemical factors	PLS	RMSE = 23.1, RE = 20.7% and r = 0.259 for post load glycemia prediction
(24)	Biomarker discovery	Metabolomics data	OPLS-DA	Formic acid, dimethylamine and galactose as novel biomarkers
(25)	Biomarker discovery	Transcriptomics data	LR	miR-223 and miR-23a as novel biomarkers
(26)	Biomarker discovery	Genomics data	LR	CC2D2B, NAT10, SIPA1, ZNF565, ZNF552, WDR35, MICALL1, CTNNB1, CLOCK, BCKDHB and TGIF2LY as novel biomarkers
(27)	Biomarker discovery	Epigenomics data	LR	cg11169102, cg21179618 and cg21620107 as novel biomarkers
(28)	Risk estimation	Physical activity questionnaire data	SL-EL	2.1 fewer cases of GDM per 100 women for moderate to vigorous intensity exercise
(29)	Disease screening	Spectrochemical data	LDA	Ac = 100%, Se = 100% and Sp = 100%
(30)	Correlation assessment	Clinical and biochemical factors	PCA	Strong correlation between maternal thyroid profile and GDM
(31)	Pharmacological treatment prediction	Mobile real-time collected data	LR	AUC = 0.8

ML, machine learning; GDM, gestational diabetes mellitus; GB, gradient boosting; LR, logistic regression; DNN, deep neural networks; RF, random forest; PLS, partial least squares; OPLS-DA, orthogonal PLS discriminant analysis; SL-EL, SuperLearner with extra learners; LDA, linear discriminant analysis; PCA, principal component analysis; AUC, area under the receiver operating characteristic curve; Se, sensitivity; Sp, specificity; Ac, accuracy; YI, Youden index; AUPR, area under the precision-recall curve; RMSE, root mean squared error; RE, relative error.

##### 4.1.1.1 ML for GDM prediction

Numerous studies that have applied ML in the context of GDM, have used it to predict this disease at early stages of pregnancy (32). Some of them have based their predictive models on a small number of variables. For example, Xiong et al. assessed hepatic, renal and coagulation function biochemical data to predict GDM at 10-19 gestational weeks (18). Univariate analysis showed that coagulation parameters differed between GDM and control women, so they combined two of them, patient prothrombin time and reference activated partial thromboplastin time, to build different ML predictive models. They achieved AUCs of 99.83% and 99.74% by light gradient boosting and SVM, respectively. Likewise, Zheng et al. used known GDM clinical and biochemical risk factors to predict it at 8-20 gestational weeks (19). By Bayesian adaptive sampling, they selected four maternal variables, maternal age, pre-pregnancy body mass index (BMI), fasting plasma glucose and triglycerides, and then used them to generate a multivariate Bayesian logistic regression model. They got an AUC of 0.766. In contrast, some articles have based their predictive models on a large number of variables. For instance, Wu et al. assessed 73 maternal clinical and biochemical variables and different ML techniques for GDM prediction before 12 gestational weeks (20). Their deep neural networks (DNN) model achieved an AUC of 0.80. Furthermore, they built a simpler model in order to facilitate clinical application. By using seven sequential feature selection chosen variables and LR they got an AUC of 0.77. Similarly, Artzi et al. used 2355 variables from EHR and gradient boosting (GB) to predict GDM before 20 gestational weeks, and obtained an AUC of 0.85 (21). They also built a simpler model to ease clinical implementation. Their nine questions based model yielded an AUC of 0.80. Interestingly, both the full and the simplified models outperformed a baseline score, which involved seven GDM known risk factors and got an AUC of 0.68.

It is worth mentioning that some papers that have sought GDM prediction, have also revealed GDM novel risk factors. That is the case of Artzi et al. study, in which the most important predictor of their full model was the prior pregnancy glucose challenge test result, a previously unreported risk factor for GDM (21). Likewise, Balani et al. used clinical data and different ML techniques to predict GDM in obese pregnant women at 14-17 gestational weeks (22). Their RF model achieved an accuracy of 77.53%, and showed that the most relevant predictor was visceral fat mass, a previously unknown risk factor for GDM.

In addition, it is interesting to notice that all the aforementioned studies reported models that allow to predict GDM, but that are restricted to do so under a particular diagnostic criteria. Recently, Mennickent et al. reported a novel strategy that overcomes that limitation (23). The authors used first trimester clinical and biochemical data and PLS to predict the very post load glycemia value that pregnant women would have at 24-28 gestational weeks. Since the predicted value can be interpreted as control or GDM with any diagnostic criteria, the prediction of GDM is no longer restricted to a particular criteria. Their best model allowed to predict the second trimester post load glycemia with a RMSE of 23.1 and a relative error of 20.7% in cross-validation analyses.

##### 4.1.1.2 ML for GDM biomarker discovery

Several studies have applied ML to search new biological markers for GDM. This has been typically done by means of omics techniques. For example, Scott et al. used **^1^** H-NMR metabolomics and 14-27 gestational weeks urine samples to find novel biomarkers for GDM (24). Their statistically significant metabolites, identified through variable importance analysis based on random variable combination, were tested for classification by orthogonal partial least squares discriminant analysis, and achieved an AUC of 0.803. The top three metabolic markers for that model were formic acid, dimethylamine and galactose, which were downregulated in GDM. Similarly, Yoffe et al. applied a targeted transcriptomics approach and 9-11 gestational weeks plasma samples to identify miRNAs that could serve as early biomarkers for GDM (25). Based on multiplex expression assays and RT-qPCR data and DESeq2 analyses, they found two differentially expressed miRNAs, miR-223 and miR-23a, which were upregulated in GDM. These miRNA markers were combined and assessed for classification by LR, and reached an AUC of 0.91. Another case is the study of Guo et al., who used a genomics strategy and 18 or less gestational weeks plasma samples to find cfDNA biosignatures that could be useful for GDM detection at early stages of pregnancy (26). Based on whole-genome sequencing and qPCR promoter profiling data, they identified 800 differentially expressed genes between GDM and control women. Eleven of those genes, CC2D2B, NAT10, SIPA1, ZNF565, ZNF552, WDR35, MICALL1, CTNNB1, CLOCK, BCKDHB and TGIF2LY, were selected by a step-wise feature selection method, and then combined and tested for classification by LR. The eleven marker based model yielded an overall accuracy of 72.1%. Likewise, Liu et al. applied an epigenomics approach to identify CpG markers for GDM (27). They used DNA methylation data from two previous studies, in which placenta samples from GDM and control mothers, and blood samples from children born in GDM and control pregnancies were analyzed. By an overlapped CpGassoc epigenome-wide association study they identified nine differentially methylated CpGs between GDM and control subjects. The LR model built with five of them revealed that the most important CpGs for GDM and control samples differentiation were cg11169102, cg21179618 and cg21620107. The combination of those three biomarkers was assessed for classification by the same ML technique, and achieved an AUC of 0.8519.

##### 4.1.1.3 Other ML applications in GDM research

Some GDM studies have used ML for other purposes, such as risk estimation, screening, correlation assessment and management. For instance, Ehrlich et al. aimed to evaluate the effect of exercise during the first trimester of pregnancy on the risk of GDM (28). Data from a pregnancy physical activity questionnaire, effected at 10-13 gestational weeks, were analyzed by different ML techniques. Their targeted maximum likelihood estimation (TMLE) and SuperLearner (SL) method with extra learners model showed that meeting or exceeding the cohort’s 75th percentile of moderate to vigorous intensity exercise reduced the risk of GDM by 2.1 fewer cases per 100 women. Another example is Bernardes-Oliveira et al. study. They intended to develop a fast and low-cost screening tool for GDM, using 9-39 gestational weeks plasma samples, attenuated total reflection Fourier-transform infrared spectroscopy and ML techniques (29). Their genetic algorithm with LDA model, which comprised ten wavenumbers mainly from lipids and proteins spectral regions, achieved an accuracy of 100%. A different case is the study of Araya et al., who meant to determine whether there was a correlation between the maternal thyroid profile and GDM (30). Using clinical and biochemical data registered at 10-14 and 24-28 gestational weeks, and PCA, they demonstrated that maternal thyroid-related hormones from the first and the second trimesters of pregnancy were strongly correlated with GDM. Finally, Velardo et al. aimed to develop a ML tool capable to improve the timeliness of GDM management (31). They used mobile health real-time collected data and different ML techniques to automatically evaluate the switch from diet-based management to pharmacological treatment. Data included blood glucose levels measured at different time points, maternal age, BMI and other GDM clinical risk factors. Their lasso feature selection LR model allowed to predict the timing of initiation of pharmacotherapy with an AUC of 0.8.

#### 4.1.2 Preeclampsia

Preeclampsia (PE) is a pregnancy syndrome that presents two different clinical scenarios, both characterized by the development of maternal hypertension from the 20th week of gestation, an alteration that persists throughout pregnancy. The first of the scenarios is characterized by a moderate form of PE, which symptoms become evident late, from 34 weeks of gestation. It is characterized by blood pressure ≥140/90 mmHg, and other symptoms that indicate liver or renal damage, thrombocytopenia or proteinuria ≥3g/24h, and by not inducing alterations on fetal growth. The second of the scenarios correspond to a severe form of PE, which symptoms become evident before 34 weeks of gestation. It is characterized by blood pressure ≥160/110 mmHg, multisystemic damage and/or proteinuria ≥5g/24h, and for being generally associated with intrauterine growth retardation (IUGR) (33). These conditions can also lead to more serious situations than PE alone, such as HELLP syndrome and eclampsia, which is a severe form of PE accompanied by seizures (34). Severe forms of PE are associated with at least two times the risk of IUGR, and fetal and neonatal death (35). The origin of PE is still unknown, however, the most accepted hypothesis indicates that the placenta does not form properly. The latter would not allow a correct flow of maternal blood towards the placenta, triggering a compensatory response that would increase blood pressure to meet the metabolic requirements of the fetus in gestation. This process would begin during the first trimester of pregnancy, producing serious effects on the mother, and affecting the fetus during the second and third trimesters of pregnancy (36). Thus, the early detection of PE, i.e. before the appearance of adverse symptoms in the mother, is necessary.

The early detection of PE has been assessed through the determination of the levels of human chorionic gonadotropin (37), anti-Müllerian hormone (38), sFlt-1 (39), the soluble form of Endoglin (40), among others, with sensitivities between 20 and 80%, and specificities between 40 and 90%. Interestingly, algorithms mediated by ML have been proposed as new strategies to predict this pathology earlier (**Table 2**). Various ML models have been developed for PE prediction using different types of variables, such as metabolites (41), proteins (42), plasma DNA (26) and circular RNA (43), but by far the most common approaches are based on maternal medical data (44–51).

**Table 2:** ML applications in PE research.

Reference	ML application	Input	ML technique	Main output
(41)	Disease prediction	Metabolomics data	LR	AUC = 0.868, Se = 75.1% and Sp = 83.0%
(42)	Disease prediction	Proteomics data	LDA	AUC = 0.96, Se = 0.90 and Sp = 0.90 for early-onset cases with maternal vascular malperfusion
(26)	Disease prediction	Genomics data	LR	AUC = 0.825, Ac = 83.0%, Se = 81.7% and Sp = 83.3%
(43)	Disease prediction	Transcriptomics data and biochemical markers	LR	AUC = 0.940, Se = 86.67% and Sp = 96.67%
(44)	Disease prediction	Biochemical markers	BPNN	Ac = 79.8%
(45)	Disease prediction	Electronic health records	Stochastic GB	AUC = 0.924, Ac = 0.973, Se = 0.603, Sp = 0.991 and DR = 0.771 for late-onset cases
(46)	Disease prediction	Electronic health records	EN	AUC = 0.89, Se = 72.3% and Sp = 91.2% for early-onset cases
(47)	Disease prediction	Clinical and biochemical factors	LR	AUC = 0.962, Se = 79.3%, Sp = 97.7%, PPV = 92% and NPV = 93.4%
(48)	Disease prediction	Clinical and biochemical factors	LR	AUC = 0.68, Se = 30.6% and Sp = 90% for early-onset cases
(49)	Disease prediction	Clinical and biochemical factors	RF	AUC = 0.976, AUPR = 0.958, Ac = 92.6%, Se = 91% and Sp = 93% for placental dysfunction-related disorders
(50)	Disease prediction	Clinical parameters	RF	AUC = 0.90, Se = 0.70, Sp = 0.89 and Pr = 0.88
(51)	Disease prediction	Ultrasound images	CNN	Se = 70.6% and Sp = 76.6% for hypertension disorders of pregnancy
(52)	Biomarker discovery	Genomics data	SVM	IL7R, IL18, CCL2, HLA-DRA, CD247, ITK, CD2, IRF8, CD48, GZMK, CCR7, HLA-DPA1, LEP, IL1B, CD8A, CD3D and GZMA as novel biomarkers
(53)	Biomarker discovery	Transcriptomics data	C4.5, AB and MLP	HTRA4, PROCR, MYCN, ERO1A, EAF1, PPP1R16B, CRH, FLNB, PIK3CB, PLAAT3, FBN2, RFLNB, and TKT as novel biomarkers
(54)	Risk estimation	Food frequency questionnaire data	SL	3.2 and 4.0 fewer cases of PE per 100 births for high density fruit and vegetable intake
(55)	Drug screening	Drug databases information	TPOTC	Estradiol, estriol, vitamins E and D, lynestrenol, mifrepristone, simvastatin, ambroxol, and some antibiotics and antiparasitics as potential drugs for PE

ML, machine learning; PE, preeclampsia; LR, logistic regression; LDA, linear discriminant analysis; BPNN, back-propagation neural networks; GB, gradient boosting; EN, elastic net; RF, random forest; CNN, convolutional neural networks; SVM, support vector machines; AB, adaptative boosting; MLP, multilayer perceptron; SL, SuperLearner; TPOTC, tree-based pipeline optimization tool classifier; AUC, area under the receiver operating characteristic curve; Se, sensitivity; Sp, specificity; Ac, accuracy; DR, detection rate; PPV, positive predictive value; NPV, negative predictive value; AUPR, area under the precision-recall curve; Pr, precision.

Some ML-based studies have aimed to predict PE before 20 weeks of pregnancy. For example, Marić et al. used clinical and biochemical maternal data and different ML techniques to predict this pregnancy complication before 16 gestational weeks. Their elastic net (EN) model achieved an AUC of 0.79 for all cases of PE, and an AUC of 0.89 for early-onset PE, showing that ML approaches can become a powerful early prediction tool for this obstetric disorder (46). Sandstrom et al. also used clinical and biochemical maternal variables and different ML techniques to predict PE, but before 15 weeks of gestation. Their LR model with 12 pre-specified variables yielded AUCs of 0.68, 0.68 and 0.67 for PE with delivery <34, <37 and ≥37 weeks of pregnancy, respectively (48). A different example is the study of Gupta et al., who aimed to predict hypertensive disorders of pregnancy, including PE, with placenta ultrasound images from the first trimester of gestation. The analysis of abnormal placental image texture with deep convolutional neural networks (CNN) achieved a sensitivity of 70.6% and a specificity of 76.6% (51). In contrast to the aforementioned articles, other ML-based studies have intended to predict PE from 20 weeks of gestation onwards. For instance, Han et al. measured 25 parameters of maternal clinical chemistry before PE clinical diagnosis, and combined them to predict this pregnancy disorder. Their back-propagation neural networks (BPNN) model, which strongest predictors were ALB, MPV, BUN, LDH and TG, displayed an accuracy of 79.8% (44). Likewise, Jhee et al. retrieved maternal data (collected between 14 and 34 weeks of pregnancy) from EHR and tested them to predict late-onset PE. Their ML models, based on decision trees (DT), naïve Bayes (NB), SVM, RF, stochastic GB, and LR reached AUCs of 0.857, 0.776, 0.573, 0.894, 0.924 and 0.806, respectively (45).

Other PE-related studies have applied ML in additional contexts, such as biomarker identification, risk estimation and drug screening. For example, Liu et al. analyzed microarray data to identify hub genes as diagnostic biomarkers of PE. Their bioinformatics approach revealed 17 differentially expressed hub genes between PE and control subjects: IL7R, IL18, CCL2, HLA-DRA, CD247, ITK, CD2, IRF8, CD48, GZMK, CCR7, HLA-DPA1, LEP, IL1B, CD8A, CD3D and GZMA. Those hub genes were combined and assessed for classification by SVM. Their model reached an AUC of 0.958 in the training set, and an AUC of 0.834 in the test set (52). Similarly, Guo et al. screened placental mRNA data to identify PE biomarkers. Their ML-based approach allowed them to select a subset of 13 mRNA features: HTRA4, PROCR, MYCN, ERO1A, EAF1, PPP1R16B, CRH, FLNB, PIK3CB, PLAAT3, FBN2, RFLNB, and TKT, which were combined and tested for PE and control subjects classification by ML. Their model, which fused three ML classifiers, C4.5, AdaBoost and multilayer perceptron, yielded an accuracy of 82.2% (53). A different case is the study of Bodnar et al., who aimed to assess the effect of fruit and vegetable intake and dietary synergy on the risk of various adverse pregnancy outcomes. Their SL with TMLE ML model revealed that high fruit and vegetable densities were associated with 3.2 and 4.0 fewer cases of PE per 100 births, respectively (54). A final example is the article of Tejera et al., who developed a ML-based strategy to identify currently existing drugs that could be repurposed for PE management. Their approach was built on pharmacological targets of drugs under clinical trial for PE, and was designed to exclude those that have shown negative effects in pregnancy. Their ML-based virtual screening identified estradiol, estriol, vitamins E and D, lynestrenol, mifrepristone, simvastatin, ambroxol, and some antibiotics and antiparasitics as potential drugs for PE treatment (55).

### 4.2 Pregnancy complications

#### 4.2.1 Perinatal death

The World Health Organization (WHO) defines perinatal deaths as those that occur from late stillbirth, i.e. after 28 weeks of gestation, up to 28 days of extra-uterine life, including late neonatal deaths (56). Worldwide, more than 5 million perinatal deaths happen every year (57). Progress in reducing the high numbers of stillbirths and neonatal deaths has been slow. Even though the rate of perinatal deaths has been lowered in developed countries, its reduction in low- and middle-income countries has been insufficient. Indeed, low- and middle-income countries present the highest rates and the slowest reduction (58, 59). The Sustainable Development Goals set by the United Nations General Assembly include to put an end to the avoidable deaths of newborns by 2030 (60), however, during 2019 there were approximately 7000 newborns deaths each day (61). These numbers highlight the necessity to implement new methods and techniques to identify high-risk pregnancies, early enough to be able to provide them personalized attention so as to improve prevention, or reduce risk and perinatal death.

##### 4.2.1.1 Stillbirth

Studies to predict pregnancies with high risk of perinatal death have been difficult due to small sample size (62). This, along with the difficulty posed by a relatively high percentage of missing data, forces researchers to look for strategies to impute missing data or lose variables to avoid biased results (63). Routinely collected perinatal records have a great potential to improve the risk assessment of perinatal death, by providing massive databases that are available for researchers to develop and test ML-based models (**Table 3**). These records are commonly composed of maternal demographic and medical history information, which can be used as predictors. The high amount of data available in these records also allows to have appropriate validation sets to assess the quality of the prediction. Koivu et al. used publicly available data obtained from the US Centers for Disease Control and Prevention, to build ML-based risk prediction models for early stillbirth, late stillbirth and preterm birth (PTB) pregnancies (64). Using only maternal demographic and medical history data (pregnancy and sexual transmitted diseases) from almost 16 million pregnancies, of which 92,753 were infant deaths, they achieved AUCs of 0.76 for early stillbirth, 0.63 for late stillbirth and 0.64 for PTB. Those results were obtained using an algorithm based on self-normalizing neural networks. An important highlight of this study is that model validation was performed using an external set from a different population, which is the strictest and most reliable type of validation, often resulting in lower performances compared to other more permissive validation methods (such as resampling methods), which are prone to overfitting.

**Table 3:** ML applications in perinatal death research.

Reference	ML application	Input	ML technique	Main output
(62)	Complication prediction	Clinical parameters	Extreme GB	AUC = 0.842, Ac = 94.71%, Se = 45.3%, Sp = 95%, PPV = 4.81%, NPV = 99.68%, +LR = 9.03 and -LR = 0.58 for stillbirth
(63)	Complication prediction	Clinical parameters	LR	AUC = 0.82 for stillbirth
(64)	Complication prediction	Clinical parameters	SNNN	AUC = 0.76, Se = 38% and Sp = 90% for early stillbirth
(65)	Complication prediction	Clinical parameters	LR	AUC = 0.872 for neonatal death
(66)	Complication prediction	Clinical parameters	RF	AUC = 0.79, Ac = 0.87, Se = 0.54, Sp = 0.88, PPV = 0.15 and NPV = 0.98
(67)	Complication prediction	Clinical parameters	DT, GB, LR, RF and SVM	AUC = 90.00%, Ac = 90.56%, Se = 91.37%, Sp = 88.10%, Pr = 88.02% and F1 = 90.58% for stillbirth before delivery and during labor
(68)	Complication prediction	Clinical parameters	MLP	AUC = 95.99%, Ac = 96.79%, Se = 86.20%, Sp = 98.37%, RMSE = 0.1702 and RRSE = 47.47% for neonatal death
(69)	Complication prediction	Clinical parameters	SL	AUC = 0.89 and U = -0.0003 for neonatal death
(70)	Complication prediction	Clinical parameters	RF	AUC = 0.922, Ac = 0.903, Se = 0.674, Sp = 0.919, PPV = 0.377, F1 = 0.477 and mean F1 = 0.712 for neonatal death
(71)	Complication prediction	Clinical parameters	ANN	AUC = 0.92, Ac = 0.86, Se = 0.86, Sp = 0.83, Pr = 0.96 and F1 = 0.91 for neonatal death

ML, machine learning; GB, gradient boosting; LR, logistic regression; SNNN, self-normalizing neural networks; RF, random forest; DT, decision tree; SVM, support vector machines; MLP, multilayer perceptron; SL, SuperLearner; ANN, artificial neural networks; AUC, area under the receiver operating characteristic curve; Ac, accuracy; Se, sensitivity; Sp, specificity; PPV, positive predictive value; NPV, negative predictive value; +LR, positive likelihood ratio; -LR, negative likelihood ratio; Pr, precision; RMSE, root mean squared error; RRSE, root relative squared error.

Using a similar approach, Malacova et al. developed stillbirth risk prediction models using different ML algorithms (62). The study population was a cohort from Western Australia, consisting in almost 1 million births, of which 5,788 were stillbirths. The variables used to build the models were a combination of maternal socio-demographic characteristics, medical history, congenital anomalies and, more importantly, current pregnancy complications, which helped to achieve the greatest sensitivity. Different models were built, since not all subjects had the same amount of information available. For all models AUC varied from 0.59 to 0.84, which suggests the importance of variable selection to achieve better performances. The best results were obtained using XGBoost, resulting in a correct prediction of 45% of all stillbirths.

Shukla et al. also performed ML-based predictive modeling for perinatal mortality, but in a wider population, a cohort of near half million pregnancies in low- and middle-income countries located in South Asia, Africa and Central America (65). They developed different models using prenatal and post-delivery variables up to two days after birth, to predict outcomes from intrapartum stillbirth and neonatal death at different time frames. The variables used included maternal, socio-demographic, and medical information along with delivery and neonatal variables (the last two for neonatal death prediction only). They observed that the prediction of perinatal deaths using just prenatal and predelivery information reached AUC values of 0.72 or less, and that the predictive accuracy of the model improved as more post-delivery variables were included. Indeed, their best results were obtained with post-delivery data, which allowed to predict neonatal deaths with an AUC value of 0.87 by LR.

Mboya et al. studied a cohort of 42,319 singleton deliveries in Tanzanian population (66) and build ML models to predict both stillbirth and neonatal death (defined as death of live births within 7 days of life) using data available in the birth registry, i.e. mainly sociodemographic characteristics. The best results were achieved using RF, NB and Boosting with an AUC of 0.79. Khatibi et al., used a two-step ensemble classifier ML-based method (including DT, GB, LR, RF and SVM) to predict both stillbirth before delivery and stillbirth during labor occurred in Iran in a population of almost 1,5 million births (67). They used a combination of maternal socio-demographic features, labor descriptors, delivery properties and clinical history of the mother and fetus, and achieved an average AUC of 0.9. Although this value is much higher than the previously discussed studies, the aim of the authors was not early prediction, but to predict stillbirth at labor-delivery instead, therefore they used variables that are not available in early prediction studies.

A common result in these studies is that gestational age and fetal height are the two most important features to discriminate livebirth from stillbirth (65–67). Some authors suggest that risk prediction models that only use demographic and medical history could be further improved with the addition of biochemical and/or biophysical variables, however to the date these approaches are yet to be explored.

##### 4.2.1.2 Neonatal death

Regarding neonatal death prediction, a recent work published in early 2021 made a systematic review on ML models used to predict neonatal mortality (72). They focused on works with a high amount of subjects (n>500 individuals) that analyzed both perinatal and neonatal factors, and excluded studies using exclusively antenatal factors, and in which neonatal mortality was not the primary outcome of study. They found eleven publications that met their criteria, among which the AUC value varied from 0.58 to 0.97. The most used ML methods were artificial neural networks (ANN), RF and LR, although the best overall model was obtained using LDA. Interestingly, from all studies reviewed in that work, only two conducted an external validation, which ensures a higher reliability. This fact also stresses the necessity of appropriate analytical methodologies and validations in future studies to ease their application by health care providers.

Other research groups, not covered in the aforementioned systematic review, have reported the prediction of neonatal death using ML-based models, with relatively high success (AUC of 95.99% for the best results) (68–70). In a different study conducted in Iran, different ML-based models were built to predict neonatal deaths in neonatal intensive care units (71). This work stands out since its models were prospectively applied and evaluated in a new cohort of neonates. Seventeen variables considered important in neonatal mortality prediction were used and different ML methods were tested, such as ANN, DT, SVM, Bayesian network and ensemble classifier. The highest AUC was achieved by the RF, SVM and ensemble models with a value of 0.98, however, when they prospectively applied the models for mortality prediction in new neonates, the best overall performance was obtained using ANN, with an AUC of 0.92, whereas the highest precision and specificity were obtained using DTs (0.97 and 0.87 respectively).

#### 4.2.2 Spontaneous abortion

Spontaneous abortion (SA) is defined as the loss of pregnancy before the 20th week of gestation (73). It is often referred also as miscarriage, but according to literature, miscarriage is considered to occur before the 24th week of gestation (74). Both situations imply a common and serious pregnancy complication that has a significant psychological impact on the mother and the family. For this reason, and due to its complicated etiology (75), SA has become a hot topic in scientific research and gynecology.

Recent advances in technology, particularly in the artificial intelligence field, have allowed the use of the increasing amount of data that can be obtained in biomedical studies to improve patients’ outcomes. This is consistent with the notion of precision medicine, that is, the need of a more personalized medicine to improve or predict the medical outcome (76), in this case, of a pregnant woman.

Interestingly, ML has been applied in the context of SA and miscarriage (**Table 4**). In 2013, Bottomley at al., developed a score based on demographic data, symptom variables and ultrasound data to predict the likelihood of a woman to have a successful pregnancy by performing a retrospective study (74). The ML method used was LR. Interestingly, the authors found that the combination of all the factors was able to provide a more accurate prediction of pregnancy viability than the obtained by analyzing the factors in a separated way, with an AUC of 0.924. This score model worked, but at that time it was not proven if it would be able to prevent miscarriage and, as the authors pointed out, the psychological morbidity associated with pregnancy loss should be integrated to the analyses. A distinct approach was made in 2019 using next generation sequencing to analyze 200 DNA samples of 100 couples presenting recurrent miscarriages (RM) (77). This work aimed to develop an algorithm based on the genetic analysis of the HLA protein codifying genes, considering the relationship of the HLA antigen sharing between couples and SA (81) in the context of immune interactions as a possible cause of SA and RM. It has been described that when the mother and the father share HLA antigens, the mother and the fetus will be homozygous for several of these loci. This issue alters the mother immunologic protection to the fetus inducing immunologic rejection and consequently SA (82). The SVM-based algorithm was able to correctly classify 67% of the total subjects, with an AUC of 0.71 and a false positive rate of 57%, which negatively affected the algorithm performance. Interestingly, this study is one of the first to predict RM probabilities in a case-by case basis, having a potential use in couple genetic counseling before conception. A different example is the study of Wu et al., who aimed to predict recurrent SA with prethrombotic state (PTS) serum biomarkers. PTS is known as one of the possible causes of SA. Wu et al. work was based on the analysis of different PTS-related proteins using multiplex array technology (78). They were able to distinguish control and affected individuals with high accuracy and precision using IL-24, exotoxin-3 and epidermal growth factor. Indeed, their DT model got an AUC of 1.000. Despite this excellent result, the cohort used for this study needs to be incremented to evaluate the real diagnostic power of this promising model.

**Table 4:** ML applications in SA research.

Reference	ML application	Input	ML technique	Main output
(74)	Complication prediction	Clinical parameters	LR	AUC = 0.924, Se = 0.922, Sp = 0.733, PPV = 84.7% and NPV = 85.4%
(77)	Complication prediction	Genomics data	SVM	AUC = 0.71, Ac = 67%, Se = 86% and Sp = 43%
(78)	Complication prediction	Proteomics data	DT	AUC = 1, Ac = 100%, Se = 100%, Sp = 100%, Kappa = 1, PPV = 1 and NPV = 1 for recurrent SA with prethrombotic state
(79)	Complication prediction	Clinical parameters	RF	AUC = 0.99, Ac = 0.99, Pr = 0.99, Re = 0.99, F1 = 0.99
(80)	Complication prediction	Electronic health records	SC	AUC = 0.909 and Ac = 89.7% for live birth

ML, machine learning; SA, spontaneous abortion; LR, logistic regression; SVM, support vector machines; DT, decision tree; RF, random forest; SC, sparse coding; AUC, area under the receiver operating characteristic curve; Se, sensitivity; Sp, specificity; PPV, positive predictive value; NPV, negative predictive value; Ac, accuracy; Pr, precision; Re, recall.

*In vitro* fertilization embryo transfer (IVF-ET) is nowadays an alternative for couples with difficulties to conceive. This procedure implies high risks of miscarriage, being psychologically stressful for couples. Therefore, it becomes necessary to find a way or system that allows the prediction of the transfer outcome, and the early detection of possible problems (83). Recently, Liu et al. developed a ML-based model with historic data obtained by transvaginal ultrasonography from females that underwent IVF-ET. The study only considered women with viable singleton and 6-12 weeks of pregnancy (79). The authors were able to predict embryonic development after transfer using six different ML-classifiers, with AUCs ranging from 0.91 to 0.97 when fetal heart rate (FHR) was included among the predictors. The most accurate prediction was obtained by RF at the 10th week after embryo transfer, with an AUC of 0.99. Other example is the article of Huang et al., who used deep learning to predict pregnancy outcomes in patients with recurrent reproductive failure (RRF), including recurrent pregnancy loss (RPL) and recurrent implantation failure (RIF). The study defined RPL as two or more SA before 20 weeks of pregnancy, and RIF as couples unable to conceive after multiple IVF-ET cycles. The authors analyzed EHR data with sparse coding, and predicted four pregnancy outcomes: biochemical pregnancy, clinical pregnancy, ongoing pregnancy and live birth. They got testing accuracies that ranged between 54.2% and 89.7% for the different pregnancy outcomes. Notably, the best model for the prediction of biochemical pregnancy was obtained with a panel of 10 endometrial immunological markers, while the best models for the other three outcomes, were obtained with a panel of 15 autoantibodies. The authors discussed that this knowledge could help clinicians to plan a more personalized diagnosis and treatment for patients with RRF (80).

#### 4.2.3 Preterm birth

The WHO defines PTB as the delivery of alive babies before 37 weeks of pregnancy are completed (56). Based on gestational age, it can be sub-categorized as: extremely preterm, before 28 weeks; very preterm, between 28 and 32 weeks; and moderate to late preterm, between 32 and 37 weeks. Most of preterm deliveries are spontaneous, although some are provider-initiated (56).

PTB is the main cause of death in children under 5 years of age worldwide. Furthermore, it has short and long-term consequences on newborns’ health, which imply a significant psychological and economic burden to families and health systems (84). The development of PTB predictive tests could be useful to identify high risk pregnancies, which could guide the healthcare personnel to offer prophylactic interventions and make antenatal management decisions (85).

ML has already been applied to develop predictive models for PTB (**Table 5**). For instance, Khatibi et al. aimed to predict spontaneous and provider-initiated PTB with data from the Iranian Maternal and Neonatal registry, which includes information of more than 1,400,0000 deliveries and 112 features. The authors used different big data ML algorithms to classify pregnant women in two steps. In the first step, all subjects were classified into term or PTB; and in the second step, the subjects classified as PTB in the first step, were then sub-classified as spontaneous or provider-initiated. Their best model, an ensemble of DT, SVM and RF, achieved a weighted average accuracy of 81%, and an AUC of 68% (86). Similarly, Belaghi et al. used first and second trimester information from the Ontario’s Better Outcomes Registry and Network database, and different ML methods to predict overall and spontaneous PTB. The investigation considered 112,963 pregnancies. For overall cases, the best models were obtained by ANN, and reached AUCs of 60.3% and 79.8% in the validation cohort at the first and second trimester, respectively. For spontaneous cases, the best results were obtained by LR, and got validation AUCs of 59.4% and 64.5% at the first and second trimester, respectively (87). A different approach was followed by Gao et al., who used EHR text data and deep learning ML methods to predict extreme PTB. Their dataset involved 10 years of EHR information from 25,689 deliveries at the Vanderbilt University Medical Center. The long short-term memory (LSTM) recurrent neural networks (RNN) ensemble model allowed to predict extreme PTB with an AUC of 0.744 in the validation cohort, greater than the obtained by LR, SVM and GB (88). This is an interesting result, although this work didn’t differentiate spontaneous from provider-initiated cases. Likewise, Zhang et al. aimed to predict PTB with continuous EHR data and LSTM. Their dataset included first and second trimester medical parameters from more than 25,000 pregnant women who received antenatal care and had vaginal delivery at the Hangzhou Women’s Hospital. Notably, the time-series deep learning technique LSTM achieved a better predictive performance than the traditional cross-sectional ML technique XGBoost, with cross-validation AUCs of 0.651 and 0.516-0.601, respectively (89).

**Table 5:** ML applications in PTB research.

Reference	ML application	Input	ML technique	Main output
(86)	Complication prediction	Clinical and biochemical factors	DT, SVM and RF	AUC = 68% and Ac = 81% for spontaneous and provider-initiated cases
(87)	Complication prediction	Clinical and biochemical factors	ANN	AUC = 79.8%, Se = 62.7%, Sp = 84.6%, PPV = 23.2% and NPV = 97.0%
(88)	Complication prediction	Electronic health records	LSTM	AUC = 0.744, Se = 0.682, Sp = 0.743 and PPV = 0.028 for extreme cases
(89)	Complication prediction	Electronic health records	LSTM	AUC = 0.651, Ac = 0.739, Se = 0.407 and Sp = 0.982
(90)	Biomarker discovery	Biochemical markers	RF	PGA2, 15DO12,14-PGJ2, BCPGE2, 13,14DHK-PGF2a, RVD1, LTE4, LTB4, linolenic acid and IL-10 as novel biomarkers
(91)	Biomarker discovery	Metabolomics data	RF	FA(17:1), FA(24:6), FA(14:2), CAR(18:2), hexanoylcarnitine, FA(14:0(Ke)), FA(26:1), raffinose, PC(18:0/16:3), FA(16:3), glycocholic acid, PC(33:4), FA(22:5), FA(14:1(Ke)), heptadecanoic acid, FA(19:1) and FA(14:1) as novel biomarkers
(92)	Biomarker discovery	Metabolomics, proteomics and transcriptomics data	RF	IL-6, IL-1RA, G-CSF, RARRES2, CCL3, ANGPTL4, PAD12, TfR, and metabolites from glutamine/glutamate metabolism, and valine/leucine/isoleucine biosynthesis pathways as novel biomarkers
(93)	Complication prediction	Electrohysterography recordings	RF	AUC = 0.999, Ac = 99.23%, Se = 98.40%, Sp = 99.76% and Pr = 95.86%
(94)	Complication prediction	Clinical parameters	KNN; RF	AUC = 1.00, Ac = 0.95, Se = 0.67, Sp = 1.00, G-means = 0.82 for PTB and the potential value of performing cervical cerclage to prolong the pregnancy; MAE = 3.521, MSE = 4.560 and R = 0.752 for timing of spontaneous delivery

ML, machine learning; PTB, preterm birth; DT, decision tree; SVM, support vector machines; RF, random forest; ANN, artificial neural networks; LSTM, long short-term memory; KNN, k-nearest neighbors; AUC, area under the receiver operating characteristic curve; Ac, accuracy; Se, sensitivity; Sp, specificity; PPV, positive predictive value; NPV, negative predictive value; Pr, precision; MAE, mean absolute error; MSE, mean squared error.

All the aforementioned studies based their predictive models on clinical and biochemical maternal information available in databases. However, other articles have assessed alternative types of data to predict PTB. Such studies are very useful to find novel biomarkers for PTB, and to propose informed hypotheses about its causes and underlying mechanisms, which are not fully understood (84, 85). For instance, Aung et al. measured an extensive set of 65 urine and plasma biomarkers, and combined them with ML to predict PTB at 26 weeks of gestation. They tested three ML methods: LR, adaptive EN and RF. The best validation results were obtained with the latter. The combination of all the biomarkers with RF yielded AUCs of 0.85 and 0.79 for overall and spontaneous PTB, respectively. Then, the authors divided the biomarkers into five groups, i.e. DNA damage markers, angiogenic factors, protein damage markers, inflammatory markers and lipid damage markers. The best predictive performances were obtained with lipid damage markers and RF, with AUCs of 0.84 and 0.79 for overall and spontaneous cases, respectively. Furthermore, the study identified the enzymatic pathway that contributed the most to that prediction: the eicosanoid lipoxygenase pathway. The combination of 15 lipoxygenase metabolites with RF got AUCs of 0.83 and 0.82 for overall and spontaneous PTB, respectively (90). Another example is the study of Chen et al., who applied untargeted LC-MS plasma metabolomics to identify metabolites that could be related to PTB, at 24-28 gestational weeks. The authors identified 17 and 16 biomarkers for overall and spontaneous cases, respectively, and tested their predictive performance with seven ML classifiers. The best results were obtained by RF, with AUCs of 0.92 and 0.89 in the testing dataset. Interestingly, most of the identified biomarkers were fatty acids, which suggests their involvement in the pathogenesis of PTB (91). Similarly, Jehan et al. performed an early pregnancy multiomics characterization of PTB. The authors applied untargeted transcriptomics and targeted proteomics on plasma samples, and untargeted metabolomics on urine specimens. They used a 2-step ML algorithm, in which a model was first trained for each omics dataset, and then combined into a final model. The integrated model achieved a cross-validation AUC of 0.83, higher than the obtained for the different omics datasets alone. The work also identified the features that were more associated with PTB: a proteomics inflammatory module, including IL-6, IL-1RA, G-CSF, RARRES2 and CCL3; and an urine metabolomic module, enriched for glutamine and glutamate metabolism, and valine, leucine and isoleucine biosynthesis pathways (92).

Some less common approaches have also been applied in the context of PTB prediction. For example, Despotovic et al. tested EHG recordings to predict PTB. They built ML models using k-nearest neighbors (KNN), SVM, RF, RF with synthetic minority oversampling technique (SMOTE), and RF with adaptative synthetic (ADASYN) sampling. Their RF-ADASYN model allowed to predict PTB at 22-25 weeks of pregnancy, with an accuracy of 99.23% and an AUC of 0.999 in cross-validation (93). A different case is the work of Rawashdeh et al., who combined 19 clinical maternal parameters with ML methods to predict PTB in a high risk cohort. They developed two different strategies to analyze their data. The first one aimed to predict whether the pregnancy would continue beyond 26 gestational weeks (the lower limit for PTB in this study) and the potential value of performing cervical cerclage to prolong the pregnancy. For this first aim, the authors tested four different classification ML methods, DT, RF, KNN and NN; solo and with SMOTE. The highest testing AUC was obtained by the KNN-SMOTE model, with a value of 1.000. The second strategy of the authors aimed to predict the timing of spontaneous delivery after cervical cerclage, an approach that wasn’t assessed in any of the previously discussed articles. For this second aim, they tested five different regression ML methods, linear regression, Gaussian process, RF, K-star and locally weighted learning. The best correlation with the actual gestational age at delivery was obtained by the RF model, with a value of 0.752 in the testing dataset. Such a regression ML model could help physicians to define prophylactic interventions timely, and reduce PTB-related perinatal morbidity and mortality (94).

#### 4.2.4 Cesarean section

Cesarean section is an effective mean to solve medical and surgical complications during dystocia and severe pregnancy disorders, and has an irreplaceable role (95). Delivery through cesarean section reduces the risk of maternal-fetal morbidity and mortality, when is medically indicated (96). Emergency cesarean section (EMCS) can be a procedure that saves lives if pregnant women experience abnormal conditions during vaginal delivery, such as fetal suffering, eclampsia or severe preeclampsia (97). Deciding to perform an EMCS is a complicated process, occurring only in specific obstetric conditions, and requires awareness and rapid assessment of the risk of the situation (98). Failure to perform EMCS on time can lead to postpartum mental disorders and other severe adverse maternal and fetal outcomes (99, 100). Recognizing an acute situation during pregnancy, labor or delivery, that can be life threatening and that could require an EMCS, is considered one of the most challenging tasks in obstetrics (101).

Visual inspection of CTG traces by obstetricians and midwives is the gold standard for monitoring the wellbeing of the fetus during antenatal care (102). One of the areas in which mathematical and computational tools for data analysis, such as ML methods, excel is in the analysis of instrumental continuous signals (**Table 6**). Several output data from instruments used in clinical diagnosis or monitoring are composed of this type of signals, in which between any two points there can be a large amount of data points, as large as allowed by the signal resolution, or even an infinite amount in the case of analog instruments. CTG traces are a great example of this type of data in obstetrics. The problem with this type of data is that its visual interpretation is highly dependent on the observer’s experience and can be strongly subjective. Most importantly, clinical decisions such as pregnancy intervention through cesarean section are made using visual inspection of CTG traces. It has been reported that the positive predictive value produced by obstetricians to anticipate negative outcomes that require cesarean section deliveries is only 30% (110). However, although human eye may fail to provide a reliable and objective interpretation, mathematical tools for pattern recognition are not subjected to the observer’s bias.

**Table 6:** ML applications in cesarean section research.

Reference	ML application	Input	ML technique	Main output
(102)	Complication prediction	Cardiotocography traces	DNN	AUC = 99%, Se = 94%, Sp = 91%, F1 = 100% and MSE = 1%
(103)	Complication prediction	Cardiotocography traces	LDA, RF and SVM	AUC = 96%, Se = 87%, Sp = 90% and MSE = 9%
(104)	Complication prediction	Cardiotocography traces	RF	AUC = 96.7%, Ac = 91.1%, Se = 90.0%, Sp = 92.2% and Pr = 92.1%
(105)	Complication prediction	Cardiotocography traces	CNN	AUC = 0.95, Ac = 94.70%, Pr = 94.71% and Re = 94.68%
(106)	Complication prediction	Electronic health records	CART	AUC = 0.7
(107)	Anesthesia dose prediction	Clinical parameters	LASSO	MSE = 0.0087 and R^2 = ^0.8070
(108)	Surgical site infection prediction	Clinical parameters and mobile images	LR	AUC = 1.0, Ac = 100%, Se = 1.0 and Sp = 1.0
(109)	Later vaginal birth prediction	Electronic health records	RF	AUC = 0.69, Ac = 70.0%, Se = 97.9% and Sp = 6.9%

ML, machine learning; DNN, deep neural networks; LDA, linear discriminant analysis; RF, random forest; SVM, support vector machines; CNN, convolutional neural networks; CART, classification and regression tree; LASSO, least absolute shrinkage and selection operator; LR, logistic regression; AUC, area under the receiver operating characteristic curve; Se, sensitivity; Sp, specificity; MSE, mean squared error; Ac, accuracy; Pr, precision; Re, recall.

Two different articles published by the same group in Liverpool have addressed the observer variability of CTG traces using a ML approach (102, 103). The authors applied signal processing techniques to extract relevant features from CTG traces and modeled the data using different ML methods, such as DNN, LDA, RF, SVM and ensemble classifiers. They were able to classify cesarean section and vaginal deliveries from CTG traces with cross-validation AUC values of 96-99%. Other study performed by an Italian research group used a similar methodology and obtained consistent results, that is, a cross-validation AUC value of 96.7% by RF (104). Likewise, a Chinese study that proposed a comparable strategy to classify normal and abnormal CTG traces reported an AUC of 0.95 by CNN in cross-validation (105). Their results demonstrate that ML methods significantly improve the prediction efficiency of necessary cesarean sections, and that their use provide a valuable decision support tool to minimize subjective interpretations of CTG traces from medical practitioners.

Besides CTG traces analysis, ML methods have been applied on EHR information to predict cesarean section and identify important variables, as well as to understand the interaction between those variables. The model developed by Clark et al. using a classification and regression tree had an AUC value of 0.7, which was considered acceptable (106). The three features that contributed the most to that model were hospital type, maternal BMI and intrapartum oxytocin dose.

Other uses of ML have been tested in the context of cesarean sections. For example, a decision-support ML-based model for assessing intrathecal hyperbaric bupivacaine dose using physical variables during cesarean section was developed, providing the anesthesiologists a new tool that gives new insights into the potential impact of controversial parameters (107). The least absolute shrinkage and selection operator regression model got a mean squared error of 0.0087. ML has also been applied to predict surgical site infection in cesarean section wounds, which is a leading cause of mortality and an important health concern in low-resource countries (108). The best model was obtained with mobile device images and LR, and achieved an AUC of 1.0. Prediction of likelihood of a successful vaginal birth after former cesarean deliveries has also been addressed using ML, which may help as a decision-making tool that could contribute to a reduction in cesarean deliveries rates (109). The EHR-based RF model reached an AUC of 0.69, better than the obtained by DT and LR.

#### 4.2.5 Fetal malformations

##### 4.2.5.1 General congenital diseases

Congenital anomalies are seen in 1–3% of the population, and approximately 60–70% of the anomalies can be diagnosed *via* ultrasonography, while the remaining 30–40% can be diagnosed after childbirth. An e-Health android application was developed by comparing the performance of nine binary ML classification models (averaged perceptron, boosted DT, Bayes point machine, decision forest, decision jungle, locally-deep SVM, LR, NN, SVM) (**Table 7**). The models were trained with the clinical dataset of 96 pregnant women and used to predict fetal anomaly status based on maternal clinical data. The decision forest model reached the best performance, with 89.5% of accuracy, 75% of F1-Score and 95% of AUC. An external validation testing with 16 users, showed that the classification algorithm accuracy was 87.5%. This estimate is enough to give a general overview of fetal health before the patient visits the physician (111).

**Table 7:** ML applications in fetal malformations research.

Reference	ML application	Input	ML technique	Main output
(111)	Complication prediction	Mobile collected data	DF	Ac = 87.5%
(112)	Complication prediction	Computed tomography images	LDA	Ac = 95.7%, Se = 92.7% and Sp = 98.9% for craniosynostosis
(113)	Complication prediction	Ultrasound images	SVM	AUC = 0.89, Ac = 88.63%, Se = 95%, Sp = 82% and +LR = 5.25 for craniosynostosis
(114)	Complication differentiation	Stereophotogrammetry images	PCA	Clear differentiation between craniosynostosis and control patients
(115)	Data acquisition	Ultrasound videos	RF	Estimation of heart position, orientation, viewing plane and cardiac phase
(116)	Data acquisition	Electrocardiography recordings	ICA and DT	Reconstruction of fetal electrocardiogram
(117)	Data acquisition	Electrocardiography recordings	SDAE	Reconstruction of fetal electrocardiogram
(118)	Data extraction	Ultrasound videos	SVM	Detection of fetal presentation and heartbeat
(119)	Data extraction	Cardiotocography recordings	EMD	Extraction of fetal heart rate
(120)	Data extraction	Electrocardiography recordings	CNN and LSTM	Extraction of fetal heart rate
(121)	Data extraction	Electrocardiography recordings	CNN and LSTM	Extraction of fetal heart rate
(122)	Data extraction	Doppler ultrasound recordings	EMD	Extraction of fetal heart rate
(123)	Complication prediction	Cardiotocography recordings	CNN	AUC = 97.82%, Ac = 98.34%, Se = 98.22%, Sp = 94.87% and QI = 96.53% for fetal acidemia caused by hypoxia
(124)	Decision making support	Cardiotocography recordings	Infant software	Identification of fetal status
(125)	Decision making support	Cardiotocography recordings	PeriCALM software	Identification of fetal status
(126)	Decision making support	Cardiotocography recordings and ultrasound measurements	Foetos software	Identification of fetal status
(127)	Complication prediction	Ultrasound measurements	NN	Ac = 95% for intrauterine growth restriction
(128)	Complication prediction	Cardiotocography recordings	SVM	Ac = 78,26%, Se = 0.78 and Sp = 0.79 for intrauterine growth restriction
(129)	Complication prediction	Ultrasound images	ANN	Ac = 91-94% for intrauterine growth restriction
(130)	Complication prediction	Echocardiography images	FINE software	Se = 98%, Sp = 93%, +LR = 14 and -LR: 0.02 for congenital heart disease
(131)	Complication prediction	Echocardiography images	CON	Ac = 99.0%, Se = 75%, Sp = 99.6%, PPV = 99% and NPV = 88.5% for congenital heart disease
(132)	Biomarker discovery	Transcriptomics data	PCA and K-means	miR-1647, miR-3064, mirR-3533, miR-6544, miR-6590, miR-6593, miR-6602, miR-6604, miR-6639, miR-6667, miR-6706, miR-6710, miR-1650, miR-1665, miR-6542, miR-6565, miR-6619 and miR-6706 as novel biomarkers for fetal alcohol spectrum disorder
(133)	Complication prediction	Clinical parameters	LR	AUC = 0.880, Se = 1.00, Sp = 0.49, PPV = 0.03 and NPV = 1.00 for macrosomia
(134)	Complication prediction	Electronic health records	LSTM	Ac = 93.3% for small, appropriate and large for gestational age
(135)	Drug teratogenicity prediction	Drug databases information	t-SNE and GB	AUC = 0.8

ML, machine learning; DF, decision forest; LDA, linear discriminant analysis; SVM, support vector machines; PCA, principal component analysis; RF, random forest; ICA, independent component analysis; DT, decision tree; SDAE, stacked denoising autoencoder; EMD, empirical mode decomposition; CNN, convolutional neural networks; LSTM, long short-term memory; NN, neural networks; ANN, artificial neural networks; CON, compound network; LR, logistic regression; t-SNE, t-distributed stochastic neighbor embedding; GB, gradient boosting; Ac, accuracy; Se, sensitivity; Sp, specificity, AUC, area under the receiver operating characteristic curve; +LR, positive likelihood ratio; QI, quality index; -LR, negative likelihood ratio; PPV, positive predictive value; NPV, negative predictive value.

##### 4.2.5.2 Craniosynostosis

Craniosynostosis is a congenital condition characterized by a premature fusion of the fetal cranial sutures, which induces one or more cranial bones in a fetal skull to join too early. Since this happens before the fetal brain is fully formed, as the brain grows, the skull can become deformed. Craniosynostosis is a common cause of pediatric skull deformities, affecting 1 of every 2000 to 2500 live births worldwide. This birth defect occurs in a predictable pattern because of localized fusions and the compensatory expansion of the cranial vault (136). It is usually detected early in life, both due to its cosmetic manifestations and functional consequences, as it can result in limited brain growth, elevated intra-cranial pressure, and respiratory and visual impairment. Early diagnosis is crucial for management, prevention of complications, and consideration for early surgical correction (112). In parallel with the growing understanding of the pathophysiology of craniosynostosis, new advances include the improvement of existing technologies such as ultrasound, and the introduction of new technologies such as ML and augmented reality (137).

Various algorithms and mathematical models have been developed to allow the computer to reliably and accurately predict specific outcomes, based on premature fusion suture input data. Using data from CT-derived measurements of cranial suture fusion, cranial deformation and curvature discrepancy, different ML methods (RF, LDA and SVM) were tested to determine the presence or absence of craniosynostosis. The best classification performance was obtained by the LDA model, with 92.7% of sensitivity, 98.9% of specificity and the probability of correctly classifying a new subject of 95.7% (112). In a different study, SVM and RF were used on ultrasound images in order to decrease the user error involved in the interpretation of craniosynostosis diagnostic imaging. They got a diagnostic accuracy of 88.63% and an AUC of 0.89 by SVM (113). Finally, PCA has proven effective in differentiating between healthy controls, scaphocephalic, and trigonocephalic patients, when applied on images obtained *via* stereophotogrammetry (114).

##### 4.2.5.3 Congenital heart disease

The incidence of congenital heart disease (CHD) has been estimated between 0.6% and 1.2% among live births (138); however, it has been reported an increased incidence of 8.3% when stillborn infants of ≥26 weeks of gestation are included (139). There could be an even higher incidence in early gestation, given spontaneous and elective pregnancy termination. A multitude of factors are associated with an increased risk of identifying CHD in the fetus, which are related to familial, maternal, or fetal conditions. The leading reason of referral for fetal cardiac evaluation is the suspicion of a structural heart abnormality on obstetric ultrasound, which results in a diagnosis of CHD in 40% to 50% of the referred fetuses. In general, subjects with risk levels exceeding ≥2% should have a detailed fetal echocardiogram by a trained examiner.

Fetal echocardiology has evolved from the description of cardiac anatomical abnormalities toward the quantitative assessment of cardiac dimensions, shape, and function. It has been demonstrated to be useful in the diagnosis and monitoring of fetuses with a compromised cardiovascular system, which may be related to several fetal conditions, such as IUGR, twin-to-twin transfusion syndrome, and CHD (140, 141). Different ultrasound approaches are currently used to evaluate fetal cardiac structure and function, including conventional 2D imaging, and M-mode and tissue Doppler imaging, among others (142). However, assessing fetal cardiac function is still challenging due to fetus involuntary movements, the small size of the heart, the high heart rate, the limited access to the fetus, and the lack of expertise in fetal echocardiography of some sonographers. After having obtained an optimal image, various measurements must be performed to extract relevant cardiac features related to remodeling and functional status. Therefore, the use of new technologies to improve the primary acquired images, or to help extract and standardize measurements is of great importance for optimal assessment of the fetal heart. ML techniques can help to optimize three different aspects of fetal echocardiology: acquisition, quantification and features extraction, and fetal diagnosis.

###### 4.2.5.3.1 Acquisition

ML-powered methods can speed up the acquisition process, decreasing the learning curve, standardizing the resulting images and increasing data quality. In such case, standardization occurs with minimal human intervention. In this regard, Bridge et al. implemented a framework for tracking key features from healthy fetal heart ultrasound videos through RF (115); and Yu et al. and Muduli et al. used independent component analysis along with a DT (116) and a stacked denoising autoencoder neural networks-based deep learning approach (117) to reconstruct fetal electrocardiography (ECG) signals from abdominal ECG recordings.

###### 4.2.5.3.2 Quantification and feature extraction

The vast majority of the research in this field focuses on automatically measuring the heartbeat. Some examples are the detection of fetal cardiac activity from maternal abdomen ultrasound videos using SVM (118), the extraction of FHR features from CTG recordings applying empirical mode decomposition (EMD) (119), the extraction of FHR from fetal ECG signals employing a combination of CNN and LSTM RNN (120, 121), and the detection of fetal heart beats from continuous Doppler ultrasound signals by EMD (122).

###### 4.2.5.3.3 Fetal diagnosis

One of the subfields in which ML has been extensively applied is the improvement of the diagnosis of fetal hypoxia or acidemia, based on CTG analyses. For example, Zhao et al. used CNN and got an AUC value of 97.82% for fetal acidemia caused by hypoxia (123). There have also been some attempts to translate these methods into clinical practice *via* the development of software that could provide additional support in the interpretation of CTG signals and, therefore, improve the assessment of fetal status. Some examples are Infant (124), PeriCALM (125) and Foetos (126).

ML has also been assessed to improve the diagnosis of IUGR, a pathology that affects about 10% of pregnancies and that has been associated with cardiac remodeling *in utero* (143). IUGR early detection models have been developed using ultrasound biometric measurements and NN (127), CTG data and SVM (128), and 2D ultrasound images and ANN (129). Such strategies got classification accuracies of 95%, 78% and 91-94%, respectively.

Finally, ML has been recently applied to improve heart diseases prenatal diagnosis. Yeo et al. presented an intelligent ML navigation method called FINE, to automatically obtain different echocardiography anatomical views of the fetal heart and identify abnormalities within the cardiac anatomy (130). Their method allowed to predict CHD with a sensitivity of 98% and a specificity of 93%. Moreover, Han et al. used an artificial intelligence algorithm based on a compound network to segment echocardiography images, and then screen for fetal CHD during pregnancy. Their method achieved an accuracy of 99.0% (131).

##### 4.2.5.4 Fetal alcohol spectrum disorder (FASD)

Gestational alcohol exposure is the most important known cause of neurodevelopmental disability, affecting nearly 5% of children in the US. It leads to complex epigenetic and transcriptomic modifications, which subsequently impair signaling pathways in neural and morphological development (144). In this regard, identifying transcriptomic mechanisms that regulate alcohol’s teratogenicity during embryonic development is crucial to understand different phenotypic outcomes, and may allow future therapeutic interventions that could mediate alcohol’s effects. In order to understand transcriptomic changes in FASD, spanning gene, exon and splicing variants, ML approaches can be used to corroborate traditional statistical methods, and to robust genomic functional studies. For example, Al-Shaer. applied PCA and K-means clustering on transcriptome sequencing (RNA-Seq) data. They identified 6857 differentially expressed exons, which represented 1251 gene IDs that deviated from baseline expression, and 18 miRNAs with significantly different expression profiles in response to alcohol. Several of those exons regulate focal adhesion, FoxO signaling, insulin signaling and Wnt signaling (132).

##### 4.2.5.5 Macrosomia

Fetal macrosomia is diagnosed when fetal growth is beyond a specific threshold, regardless of the gestational age. In developed countries, the most used threshold is a weight above 4,000 g (145). Macrosomia is associated with an increased risk of several maternal and newborn delivery complications, like shoulder dystocia, brachial plexus injury, asphyxia, prolonged labor, postpartum hemorrhage, and laceration of the anal sphincter (146). Predicting macrosomia is important for making decisions about induction or cesarean delivery before the start of labor. For example, Shigemi et al. developed LR and RF ML models to predict macrosomia using maternal clinical parameters. The generated LR risk scoring system allowed to determine the association of each predictor with macrosomia, and achieved an AUC value of 0.880 (133). Likewise, Tao et al. tested different ML techniques to predict fetal birthweight from EHR data. They considered three categorical outcomes: small for gestational age (SGA), appropriate for gestational age (AGA) and large for gestational age (LGA). SGA was defined as birthweight lower than 2,500 g; AGA as birthweight between 2,500 and 4,000 g; and LGA as birthweight greater than 4,000 g. Remarkably, the time-series deep learning technique LSTM achieved a classification accuracy of 93.3%, outperforming the traditional cross-sectional ML techniques LR, BPNN, CNN and RF (134).

##### 4.2.5.6 Teratogenicity

Teratogenicity is the most serious manifestation of iatrogenic fetal toxicity. Developing fetuses are especially sensitive to chemical exposures. Teratogens lead to fetal malformation and are implicated in lifelong physical and/or mental disabilities (135). Teratogenicity scoring for small molecules is unsystematic, and is performed outside the clinical environment (147). Moreover, prescribing behavior for gravid patients is based on limited human data and conflicting cases of adverse outcomes, due to the exclusion of pregnant populations from randomized controlled trials (148). Using unsupervised t-distributed stochastic neighbor embedding and supervised GB ML methods, Challa et al. demonstrated that small molecule drug structure is a good predictor of teratogenicity. The application of such methods also allowed to discover relationships between chemical functionalities within drugs prescribable in pregnancy and existing teratogenicity information. Three chemical functionalities that predispose a drug towards increased teratogenicity and two moieties with potentially protective effects were discovered. The ML algorithm predicted three clinically relevant classes of teratogenicity with an AUC of 0.8, and nearly double the predictive accuracy of a blind control for the same task, suggesting a successful modeling (135).

## 5 ML in pregnancy diseases and complications: current state and future challenges

### 5.1 Current state

ML has been widely applied in all the seven subjects considered in this review: gestational diabetes mellitus, preeclampsia, perinatal death, spontaneous abortion, preterm birth, cesarean section, and fetal malformations. The applications are varied, including early detection, alternative screening, biomarker discovery, risk estimation, correlation assessment, pharmacological treatment prediction, drug screening, data acquisition, data extraction, among others. We observed that the most common ML use is the prediction of perinatal diseases or complications. This is in line with what was described in two recent reviews on ML and pregnancy care. The scoping review of Abuelezz et al. explored the contribution of artificial intelligence in pregnancy, and categorized the applications in “prediction of pregnancy disorders/complications”, “treatment and management” and “assist with patients’ safety outcome”. 75% of the reviewed studies fell into the first category (4). Likewise, the systematic review of Islam et al. dug into the use of ML to predict pregnancy outcomes. They categorized the reviewed articles according to their scope: “predicting pregnancy risks/complications”, “exploring pregnancy factors”, “predicting mode of delivery”, “predicting outcome of IVF treatment”, “predicting labor outcome” and “comparing two birth weight groups”. The most common was the first category, with a frequency of 35% (5). Furthermore, we noted that the number of studies employing ML in pregnancy has increased over time, with most of the reviewed articles being published in the last five years. This tendency was also identified by previous reviews in the field (4, 5, 149).

Depending on the type of data available, different ML methods are preferred for studying pregnancy-related alterations. When the data available come from medical records, the information available is rich in socio-demographic characteristics, medical history variables and anthropometric measurements. We observed that when this is the case, the researchers usually have a massive amount of data (patients) available, obtained from the aforementioned medical records, to train the ML model. In this scenario, the most used ML methods correspond to non-linear methods, such as SVM, NN, DT, ensemble methods, etc. This could be explained by the fact that correlations between this type of data and the diseases or complications we focused on in this review are complex, not directly or linearly correlated. Non-linear and non-parametrical methods seem to be more suitable in such scenario, in which data is affected by a higher amount of variability and uncertainty. This is especially true when data from medical questionnaires and other surveys are used, in which the answers and values obtained thereof are highly dependent on the patient’s perception. Appropriate variable selection and validation of the models is perhaps even more important in those cases. In several studies reviewed in this work, the authors used some level of validation to test their models, and therefore, the accuracies they reported demonstrate a certain relationship between the data used and the pathology studied, even though that relationship is not necessarily linear. Therefore, it is possible to obtain adequate ML models to study adverse perinatal outcomes from data already available. This adds value to currently existing medical records databases.

A fundamental precept in data science is that, in order to predict a property (e.g. a pathology, or the concentration of a particular biomarker) the data must contain information related to that property, and the stronger the correlation, the better the performance of the model. In this regard, it has been suggested that prediction models could be improved when using biochemical or biophysical variables (64). This type of data is less affected by human bias and is more directly related to the physiology of an individual, or the pathophysiology of a disease. Most variables of this type correspond to biochemical analytes or ultrasonography parameters. In this scenario, the type of variables used are not too different from the data used in chemical, environmental or pharmaceutical sciences. Analytical chemists have been successfully using chemometrics (i.e. ML applied in chemistry) for several decades to extract relevant information from chemical data, to find correlations or predict a sample property. In essence, the exercise to identify the origin of certain wine from its metal profile, an example of a common application of chemometrics in analytical chemistry, would be no different than predicting a pathology based on the characteristic multivariate pattern of a blood biochemical profile. Likewise, biophysical variables such as the continual recording of FHR through CTG are very similar to the graphical outputs obtained from the analytical instruments used in chemistry (e.g. chromatogram or spectrogram), in the sense that an analytical signal is continuously recorded from an instrument. Therefore, the robust chemometrical platform used in analytical chemistry for the analysis of this type of data could also be exploited in biomedical science. In chemometrics, the most used methods are linear, i.e. are based on linear combinations of the original variables, with which they find hidden correlations that can be used to predict a particular property. Methods such as PCA, partial least squares regression, soft independent modeling of class analogies, discriminant analysis, or variations of them, are among the most used in chemistry (6, 7). These methods are more intuitive than the non-linear methods mentioned before. Furthermore, they usually provide valuable information about the importance or weight of the variables on the prediction of a certain property, as well as variable-variable and variable-sample relationships, which are some of the reasons they are preferred in chemical analysis. Curiously, in this review we observed that these methods are not common in pregnancy-related applications, where non-linear methods are the trend and LR seems to be almost the only linear method chosen. This observation is consistent with the systematic review and meta-analysis of Sufriyana et al., who found that the most common ML techniques in prognostic prediction studies for pregnancy care are LR (64.8%) and ANN (14.1%) (150). As clinical chemistry can be considered as a type of analytical chemistry, a more widespread application in biomedicine of the linear ML methods used in chemistry could be highly beneficial, whenever biochemical data is available.

### 5.2 Future challenges

It is difficult to think of a field of knowledge in which ML has not been applied. Consequently, it is quite challenging to be innovative regarding the use of ML in the context of pregnancy diseases and complications. An aspect that could be improved is data management, for example by automating their recording, storage and update in both medical and research settings. The later could ease data extraction, analysis and posterior interpretation. Even though EHR are common in developed countries, they are not frequent in low- or middle- income countries (151, 152). Therefore, the spread of EHR and their adaptation to different realities is an important task for the scientific community in the near future. Moreover, it is necessary to adapt ML applications to the emerging technologies in biomedical sciences, with which novel and more complex types of data can be obtained (153). This could be employed not only to develop more accurate predictive models, but also to find new biomarkers that could help to better understand the pathophysiology of a particular disease or complication, which could in turn lead to an improvement in its prevention, management or treatment. Furthermore, although there are a lot of published ML models aiming to improve maternal and fetal health, many of them have never been validated, nor subjected to impact analysis. This translational issue was also identified by a recent systematic review on ML-based clinical decision support systems in the context of pregnancy care (154). To be considered suitable for clinical implementation, ML models have to exhibit a good predictive performance in both internal and external validation, and also prove to foster positive changes in medical settings (e.g. improve patients outcomes, reduce management costs, etc.) without impairing care quality and patient satisfaction (14). Hence, besides developing new ML tools in the field of pregnancy alterations, it is necessary to carry out studies to test the already published models in different populations and healthcare facilities. This would allow to know if it is really worthwhile to implement them in clinical practice. To perform such studies is a demanding task, since the recruitment and follow-up of large cohorts of subjects require a very well-coordinated multidisciplinary work, and both time and financial resources are spent. However, it is the only way to lead ML models closer to real medical applications.

Pregnancy lasts only nine months, and the first three have been proposed as the ideal time frame for the early detection, treatment and management of gestational alterations (155–157). This window of time is narrow, but represents a great opportunity to exploit all the advantages that are associated with the use of ML, i.e. finishing complex assignments rapidly, dealing with multiple tasks efficiently, and predicting short- and long- term outcomes accurately (158, 159). Indeed, this review widely demonstrates that ML methods have a great potential to be applied in such a context, and to contribute to reducing the impact of pregnancy diseases and complications on maternal and fetal health.

### 5.3 Strengths of this review

This review is not restricted to a particular ML application on pregnancy diseases and complications. There are a couple of recent systematic reviews that are similar to our work, however they focus on a specific ML application in the field of pregnancy care, such as the screening of adverse perinatal outcomes (160) and the prediction of perinatal complications (149). In contrast, this review covers the wide variety of applications that ML may have on maternal and fetal health, including not only the screening or prediction of perinatal alterations, but also biomarker discovery, risk estimation, correlation assessment, pharmacological treatment prediction, drug screening, data acquisition, data extraction, among others, in the context of such alterations. Moreover, this review has a marked clinical focus. There are some recent narrative and systematic reviews that describe different pregnancy-related ML applications, but their emphases are on the applications themselves, and not on specific perinatal pathologies (4, 5, 161, 162). On the contrary, this review focuses on particular diseases and complications, and gives a broad overview of ML applications for each, which allows to visualize how much ML has penetrated into specific areas of the field of obstetrics and gynecology. Finally, this review covers a considerable body of literature. Most of the reviews found in literature regarding ML and perinatal care include a small number of references (4, 5, 149, 160, 162). Contrarily, this review comprises an important number of scientific articles, which ensures giving a comprehensive overview of the state of art regarding the use of ML in the context of pregnancy diseases and complications.

### 5.4 Limitations of this review

Due to the narrative nature of this review, the search and selection of articles was not performed by means of a systematic protocol. Hence, it could be subjected to bias. In addition, this review was restricted to seven selected pregnancy diseases and complications, and English-written articles. Hence, we may have missed some promising ML applications in the field of maternal and fetal health.

## 6 Conclusion

The use of ML methods in the context of pregnancy diseases and complications is fairly recent, and is becoming increasingly popular. The applications are varied, and go beyond diagnosis. Indeed, ML has been used to improve the management, treatment, and also the understanding of the pathophysiological mechanisms underlying different perinatal alterations. Facing the challenges that come with working with different types of data, the handling of increasingly large amounts of information, the development of emerging technologies, and the need of translational studies, it is expected that the use of ML methods continue growing in the field of obstetrics and gynecology in the coming years.

## Author contributions

DM, AR, CO, CR, EC, AE-S, JA-R, EG-G, and JA tabulated literature information; DM and JA wrote the manuscript; DM, CA, AD, EG-G, and JA improved the manuscript. All authors contributed to the article and approved the submitted version.
